# National Thanksgiving and How to Show It

**Published:** 1918-12-28

**Authors:** 


### NATIONAL THANKSGIVING AND HOW TO SHOW IT.

Now that the armistice has come, people are beginning to discover that peace has its disasters no less terrible in their effects than those of war. In what spirit are we to confront them? A dangerous assertion was widely made in the earliest days of our anxiety, the assertion that the war had produced a change of heart. People who doubted the truth of this assertion at the time incurred unpopularity. They were thought to be encouraging panic. Whether they were right or wrong shall be proved now that the fighting is over. For a change of heart is a permanent change; that is how it is to be distinguished from a fit of terror. In the first flush of excitement most of us were ready to attempt things which we should not have attempted before. Attempts were made to rise to an emergency. Such attempts, even when sincere, cannot justly be called heroic, for heroism is the display of exceptionally fine character. It is not shown in a piece of daredevilry. It is not a flash in the pan. Since few of us like to be called heroes, it may be well to look behind the emergency which startled us into unexpected activity, for some force which will guide us through the perils of a return to peace. It is idle to assert that the war has changed us. People are not reformed from without. The crisis of the life of a man is not the moment which is apparent to the crowd. As one of our poets has said:—

> The day of days was not the day;
> That went before, or was postponed;
> The night death took our lamp away
> Was not the night on which we groan'd.
> I drew my bride, beneath the moon,
> Across my threshold; happy hour!
> But, ah, the walk that afternoon
> We saw the water-flags in flower!

If this be true, and great poets are supposed to be fine observers, we must look not so much to the shock and fever of war to provide us with the "new men" whom we need for the problems of a return to peace, so much as to those whom the war did not shock into attention, but found and has left the men of honour they always were. The man whom only war can teach is practically unteachable, and if we look to such we shall learn this to our cost. Where, then, in the principles of men of honour, is that, the most simple, which was acknowledged before the war, adapted to the war, and therefore capable of surviving the war? The answer is the principle of personal service.

Now, though personal service is a duty, and implies in its performer an attitude of mind with respect to the whole of life, it is a duty more apparent in the wards of a voluntary hospital than elsewhere. This is because the appeal of pain to our sympathy is the most primitive of all appeals. In the voluntary hospitals the need of the sick is accepted as the foundation of the duty of service to others, but there come within its spell many who never witness the ordinary scenes of hospital life. The innumerable supporters of the voluntary hospitals come to their assistance because they have learnt that each of us is a trustee for his own health, and that it is an acceptable act of thanksgiving, while we are well, to do something for the sick among whom accident or illness at some time or other may cast us. This sense of trusteeship, and of responsibility, form what is often called the gospel of personal service. To such as understood it, the war came only as the crudest example of its truth. To such as held it, the war caused no awakening. They were awake before. This doubtless explains why the voluntary hospitals adapted themselves more easily to the conditions of war than any other institutions. The war may have come as a shock to the War Office. It was part of the day's work to the voluntary hospitals. Those who took an active part in maintaining the voluntary system obeyed the duty of personal service through their daily work, which indeed was inspired by it. These men, working quietly, patriotically, and unseen before the war, faced its occurrence undismayed, saw the nation through with their part of its burden, and made the British Red Cross the most universal and efficient of all our organisations, except the British Fleet. It is, therefore, fair to claim that the voluntary hospitals are guardians not only of the sick citizens, but of English public spirit and true citizenship. Consequently the event of war and the return to peace are problems which leave them equally undismayed, and find them on either occasion doing their duty. It is to that spirit alone that the future can be entrusted without fear of revolution or disorder. It remains to show why in the future, whatever it will bring, the voluntary hospitals have to take an important part.

Their imperfection was revealed in a serious shortage of beds, so that to some extent the needs of civilians had to be sacrificed to the needs of the soldiers. Consequently their first duty will be to rebuild considerably. For that purpose funds will be required, and we who believe in the spirit of service which they foster, and are grateful for the great work which they have performed for the wounded, should make our thank-offering this Christmas a contribution to their building funds. Here is an example of solid reconstruction. Here is an instance of rebuilding which appeals to the imagination of all. Such funds as have been raised during the war have been mainly increases of income necessitated by the rise in prices of all the commodities. If increased incomes can be maintained there will be some annual reserve in hand to maintain the new wards which must be built. It is our duty to supply the capital necessary for the new buildings which are urgently required. Their cost must be the thank-offering of the British public, so that the voluntary principle which preserved the ideal of duty through the long summer of peace, and bore calmly the demands of the dark months of devastation, may be maintained to relieve the strain of reconstruction. Christmas is the festival of the birth of the Prince of Peace, and at this renewal of His blessings let us turn with generosity to the hospitals which peace and war alike find pursuing His business in works of mercy and self-dedication to the needs of others.

